# Current Practice of Heart Donor Evaluation in Germany: Multivariable Risk Factor Analysis Confirms Practicability of Guidelines

**DOI:** 10.1155/2013/701854

**Published:** 2013-09-30

**Authors:** Sylke Ruth Zeissig, Carl-Ludwig Fischer-Froehlich, Frank Polster, Nils R. Fruehauf, Guenter Kirste, Irene Schmidtmann

**Affiliations:** ^1^Institut für Medizinische Biometrie, Epidemiologie und Informatik (IMBEI), Universitätsmedizin der Johannes Gutenberg-Universität Mainz, Obere Zahlbacher Straße 69, 55131 Mainz, Germany; ^2^Deutsche Stiftung Organtransplantation (DSO), Region Baden-Württemberg, Kriegerstraße 6, 70192 Stuttgart, Germany; ^3^Deutsche Gesellschaft für Gewebespende gGmbH, Feodor Lynen Straße 21, 30625 Hannover, Germany; ^4^Landesärztekammer Niedersachsen, Berliner Allee 20, 30175 Hannover, Germany

## Abstract

*Background*. Organ shortage has liberalised the acceptance criteria of grafts for heart transplantation, but which donor characteristics ultimately influence the decision to perform transplantation? For the first time this was evaluated using real-time donor data from the German organ procurement organization (DSO). Observed associations are discussed with regard to international recommendations and guidelines. *Methods*. 5291 German donors (2006–2010) were formally eligible for heart donation. In logistic regression models 160 donor parameters were evaluated to assess their influence on using grafts for transplantation (random split of cases: 2/3 study sample, 1/3 validation sample). *Results*. Successful procurement was determined by low donor age (OR 0.87 per year; 95% CI [0.85–0.89], *P* < 0.0001), large donor height (OR 1.04 per cm; 95% CI [1.02–1.06], *P* < 0.0001), exclusion of impaired left ventricular function or wall motion (OR 0.01; 95% CI [0.002–0.036], *P* < 0.0001), arrhythmia (OR 0.05; 95% CI [0.009–0.260], *P* = 0.0004), and of severe coronary artery disease (OR 0.003; 95% CI [<0.001–0.01], *P* < 0.0001). Donor characteristics differed between cases where the procedure was aborted without and with allocation initiated via Eurotransplant.

## 1. Introduction

According to the International Society for Heart and Lung Transplantation (ISHLT), worldwide about 5000 heart transplants are carried out yearly [1]. In Germany in 2011, 366 heart transplantations were performed at 22 hospitals; however, 695 patients were placed on the waiting list in the same year [2]. Due to the existing organ shortage, the acceptance criteria for donor hearts have been increasingly liberalised. Numerous recommendations on the evaluation of potential donor hearts have been published [3–13]. But what about their implementation in daily work of transplantation coordinators?

This study evaluates for the first time which factors ultimately influence the decision to use a graft for heart transplantation or not by using real-time data of donation procedures provided for allocation and decisions at recipient center level. The actual decision pathways in the donation process are discussed with reference to the published recommendations and guidelines. At different levels they are more or less triggered by the organ procurement organization (OPO) or recipient centers.

Optimal evaluation of potential donor hearts is a cost-intensive purpose, including special cardiac diagnostic procedures like coronary angiography. Strategies to avoid a waste of resources have to be discussed in health policy.

## 2. Methods

The national OPO, the German Foundation for Organ Transplantation (DSO), coordinates all organ donations in Germany. Only allocation is outsourced by law to the international organ exchange organization Eurotransplant (ET). In total, 15 484 pseudonymous DSO data records from potential or realised donors from January 2006 to December 2010 are available. Over 160 donor variables were analysed. All free text results of imaging examinations (ECG, echocardiography, and coronary angiography) were assessed by three experts in a review process; the results were classified according to the risk estimates published by Grauhan et al. on behalf of the national transplantation society (Deutsche Transplantationsgesellschaft (DTGs)) [4]. The statistical analysis was conducted using SAS version 9.2 (SAS Institute, Cary, NC).

**Figure 1:**
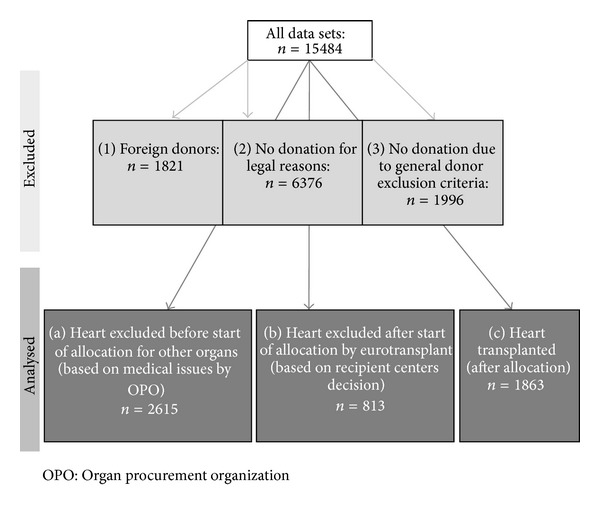
Flow chart of the analysed DSO data records from the years 2006–2010.

Because this paper aims to evaluate the medical reasons for deciding for or against realisation of heart transplantation from potential donors, all cases where heart donation was impossible due to legal reasons (e.g., missing consent; *n* = 6376) were excluded. Records of donations from outside of Germany with grafts transplanted in the country had to be excluded because of lack of medical data as the DSO was not entitled to records beyond administrative issues (*n* = 1821). Cases were excluded if the entire donation process had been terminated before registration of the donor with ET for allocation of at least one organ, because in these cases only limited data about medical reasons were documented, which is in accordance with the rules of national personal data protection laws (*n* = 1996) (Figure 1). The remaining 5291 cases were randomly divided into a training sample, containing about 2/3 of the cases (*n* = 3536), and a validation sample (*n* = 1755). First, the dependency of heart transplantation on each potentially influencing variable in the training sample was investigated using univariate analyses. We used the *χ* ^2^ test, the Jonckheere Terpstra test, and the measure of association Gamma; quantitative characteristics were evaluated using empirical distribution curves and the Kruskal Wallis test.

The multivariate analysis was conducted using logistic regression. The following criteria were used to determine which of the 160 investigated variables were included in the multivariate analysis (see Table S1 in Supplementary Material available online at http://dx.doi.org/10.1155/2013/701854):

1. inclusion criteria:
  - medical relevance, frequency of quotation in the literature;
  - OR, width of confidence interval, and *P* value of score test ≤0.2 (univariate logistic regression);
2. exclusion criteria:
  - high number of missing values;
  - possible interactions and correlations with other variables (in case of correlation, the determining factor for inclusion was medical relevance and number of missing values);
  - surrogates of variables already included in the model;
  - extremely rare comorbidities (positive findings <0.5%).

The 41 variables included in the model are shown in Table S1 (Supplementary Material). To select explanatory variables during model building, forward stepwise selection was performed. To validate the results and as a sensitivity analysis, a backward selection was also applied. Model testing was done using the validation sample. To quantify the ability of the logistic regression model to predict results, the proportion of concordant pairs (pairs where observed and predicted results (heart transplantation yes/no) agree) was determined. 

Three different decision levels exist regarding the realising of heart transplantation from a potential donor:

 Level A: Registration of the donor with ET for allocation of at least one organ with the heart excluded for medical reasons based on data from the OPO.
 Level B: Registration of the donor with ET for allocation including the heart, but heart transplantation not realised more or less based on decisions by recipient centers.
 Level C: Registration of the donor with ET for allocation including the heart and heart transplantation realised.

The results of multivariate analysis comparing Level A to C and Level B to C separately are described. Data from the training sample are shown (significance level 5%, odds ratio [95% CI] for realising heart transplantation from a potential donor).

## 3. Results

**Figure 2:**
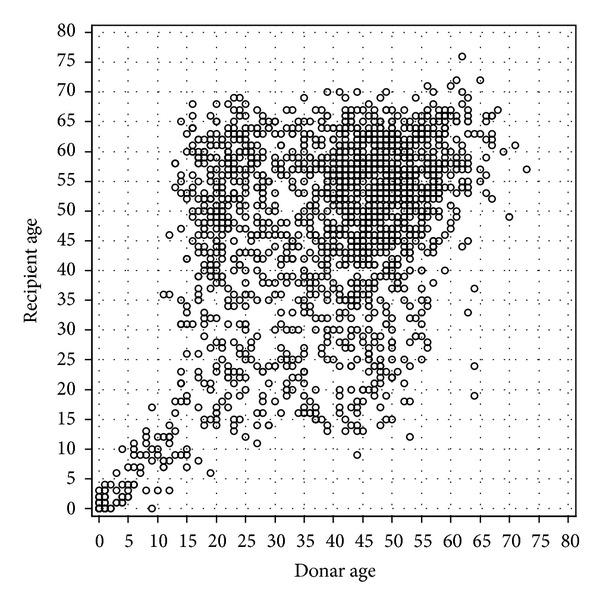
Correlation between donor age (horizontal-axis) and recipient age (vertical-axis).

From 2006 to 2010, 1863 heart transplants were realised with grafts procured from donors in Germany (Table 1). The gender was matched between donor and recipient in 1272 cases (68.3%). Most recipients were men (*n* = 1425; 76.5%), as were the majority of the donors (*n* = 1045; 56.1%). The recipients were on average 7.5 years older than the donors. Up to 15 years of age donors and recipients were age-matched, probably due to size matching. Recipients older than 70 years received organs from elderly donors. Between these two margins, the donor and recipient ages were not well matched (Figure 2). In univariate analysis remarkable differences existed in donor parameters when comparing cases of Level A with cases of Level C, but not for comparing cases of Level B with cases of Level A. Levels A and C differed most distinctly in heart-specific examinations (ECG, echocardiography, and coronary angiography), age, catecholamine requirements, weight, body mass index (BMI), length of stay in the intensive care unit, hemoglobin count, and prothrombin time. More frequently, heart enzymes (Troponin I, Troponin T, CK-MB) were elevated in cases of Level A compared to Levels B and C. This is also true for unacceptable risk factors after quantitative determination of left ventricular function (e.g., ejection fraction), left ventricular hypertrophy (e.g., intraventricular septum), and other morphologies.

The results of multivariate analysis comparing donor characteristics of cases in Level A with those of Level C as well as in Level B with Level C are summarised in Table 2. 

Cases deemed unsuitable for heart transplantation at Level A had fewer cardiac diagnostics than cases excluded at later stages. For example; only for 40% of patients in Level A electrocardiography was documented, and only for 29% echocardiography was performed, whereas results of those cardiac diagnostic procedures were available in Level C for 94.8% (ECG) and 96.6% (electrocardiography), respectively.

Different decision patterns are described. On the one hand, there are the coordinators, who decide whether or not a potential donor should be allocated by ET (Level A), and on the other hand there are factors decisive for the final result (Level B) that exist between the start of allocation by ET and the heart surgeon's final judgment of the donor organ. Varying odds ratios of some influencing variables between Levels A and B (Table 2) indicate a selection process in which certain donor characteristics clearly lead to the exclusion of a potential donor heart early in the process or affect this in some other ways so that the variables are no longer significant at the next level of decision making. Examples are gender (female versus male; A: 1.90, 95% CI: 1.11–3.32, *P* = 0.02; B: 1.08, 95% CI: 0.77–1.51, *P* = 0.67), type of brain damage (secondary versus traumatic; A: 0.21, 95% CI: 0.09–0.50; *P* = 0.0004; B: 0.59, 95% CI: 0.34–1.03, *P* = 0.06), and length of time spent in the intensive care unit (per day; A: 1.40, 95% CI: 1.21–1.61, *P* < 0.0001; B: 0.99, 95% CI: 0.90–1.09, *P* = 0.87). 

All the variables that were selected in the forward selection process in the training sample were remodeled using the validation sample. The results are shown in (Tables S2 and S3, Supplementary Material). All models showed a high congruence in the proportion of concordant pairs. In particular variables with a significant impact and not coding the category of missing values showed similar odds ratios and *P* values. 

Recovery of a heart for heart transplantation was limited at Level A by donor age, height, gender, secondary brain lesions, length of stay in ICU, need for high inotropic support, severe functional abnormalities detected by ECG or echocardiography, and evidence of coronary heart disease (Table 2). At Level B the impact of some of these variables changed or even disappeared (e.g., inotropic support).

## 4. Discussion

In summary, donor age, body size, a coronary artery disease diagnosis, and the results of using the German Transplant Society's parameter catalog for judging donor hearts [3, 4] regarding ECG and echocardiography are influencing factors for the decision to procure a donor heart for heart transplantation or not. In terms of cardiac diagnostics, left ventricular function and the presence of possible wall motion abnormalities in the echo are particularly important. Of slightly less importance are other current heart conditions in the donor: left ventricular hypertrophy and valvular deficiency. Infections in the donor, laboratory parameters, type of brain damage, reanimation, days spend in intensive care, and the need for catecholamines were less important or inconsistent regarding the impact on successful procurement for heart transplantation. 

### 4.1. Adherence to Existing Guidelines

High *donor age* is a known risk factor limiting survival after heart transplantation [14, 15]. In this study, donor age was a predisposing factor for using a graft for heart transplantation (adjusted OR per year for Level A: 0.87, 95% CI: 0.85–0.89, *P* < 0.0001; for Level B: 0.95, 95% CI: 0.94–0.96, *P* < 0.0001). Greater *donor size* was related to a higher rate of grafts used for heart transplantation (adjusted OR: per cm body height for Level A: 1.04, 95% CI: 1.02–1.06, *P* < 0.0001, which means that with each 1 cm increase in a donor's height, the chances that a heart is used is increased by 4%; for Level B: 1.02, 95% CI: 1.02–1.03, *P* < 0.0001). This can be explained by the need to avoid heart transplantation with an undersized graft [7].

The diagnosis of *coronary heart disease* (CHD) limits the use of grafts for heart transplantation [16]. The adjusted odds ratio in Level A for diagnosis of CHD was 0.003 (95% CI: 0.001–0.01, *P* < 0.0001), thereby virtually excluding transplantation. The ISHLT guideline recommends not using grafts with obstruction of the main coronary arteries [6]. But according to Hauptmann et al., CHD onset without hemodynamic-relevant stenosis or with only a local stenosis should not exclude transplantation [17]. Ultimately, the consequences of an abnormal coronary angiography result (e.g., rejecting the graft or simultaneous coronary surgery/PTCA/stent at implantation) must be decided on an individual basis [4]. Of the known risk factors for CHD, only *arterial hypertension* was a significant risk factor at Level A (OR: 0.36, 95% CI: 0.20–0.63, *P* = 0.0003). Generally arterial hypertension may result in *left ventricular hypertrophy *(LVH). Severe LVH detected by echocardiography was associated with an increased risk of not using the graft for heart transplantation at Level A (OR: 0.20, 95% CI: 0.09–0.49, *P* = 0.0004) and Level B (OR: 0.43, 95% CI: 0.21–0.89, *P* = 0.0154). According to the ISHLT, using grafts with LVH appears to be appropriate if an ECG shows no evidence of LVH, and the thickness of the left ventricular wall does not exceed 14 mm [6]. Relevant changes in *heart valve morphology* (e.g., stenosis or insufficiency > grade 1) were also a risk factor for not using the graft (Level A: OR 0.04, 95% CI: 0.01–0.11, *P* < 0.0001 and Level B: OR 0.32, 95% CI: 0.14–0.73, *P* = 0.0072). According to the ISHLT, there is no reason to prevent the use of donor hearts with valvular insufficiencies, especially since intra-operative corrections are possible [6, 18, 19]. Relevant hypokinesia or regional *wall motion abnormalities* detected by echocardiography were a highly significant factor for excluding a heart from donation (Table 2). The same applied to severely reduced *global left ventricular function* (LVF) when compared to normal function (Level A: OR: 0.007, 95% CI: 0.002–0.036, *P* < 0.0001 and Level B: OR: 0.083, 95% CI: 0.019–0.357, *P* = 0.0008). Hearts with an insignificant reduction in LVF are used as frequently as hearts with normal LVF; this is not listed as a reason to exclude a donor heart in the existing guidelines. However, there is considerable hesitation to transplant hearts that have abnormal wall motion, such as regional akinesia or global hypokinesia. Whether these abnormal regional wall movements are correlated with consequential damage due to coronary sclerosis which entails irreversible damage or are the result of temporary neurocardiac dysfunction which entails reversible damage [20] cannot be determined by the echocardiogram results. This can only be established by taking the other risk factors into consideration as well or within the context of a diagnostic coronary angiography. Potential donor-derived transmissible *infections* like meningitis, pneumonia, cytomegalovirus, toxoplasmosis, and recovery from sepsis were insignificant risk factors in multivariate analysis. This is in agreement with the results of other authors [21, 22]. However, under certain conditions (e.g., effective bacteria-specific antibiosis, repeated negative blood cultures, and no evidence of endocarditis), the ISHLT sees no contraindication for using a heart with normal myocardial function [6]. Regarding the *laboratory parameters*, the heart enzymes are considered as relevant for donor heart selection [4]. However in this analysis, CK (creatinine kinase), CK-MB (Muscle-Brain type CK), and LDH (lactate dehydrogenase) were not significant or were not consistently significant in all the analysed groups. Only the Troponin values had a clear influence on whether a transplantation took place or not (strongly elevated Troponin versus normal (≤0.1 *μ*g/L), Level A adjusted OR: 0.19, 95% CI: 0.07–0.54, *P* = 0.0018). This observation underlines Grauhan et al.'s hypothesis that ascertaining the Troponin level is of greater importance than the CK-MB levels, which can also be elevated due to cerebral damage [4].

Regarding the need for *catecholamines*, the ISHLT recommends rejecting a potential donor heart if there is a great need for inotropic substances (dopamine 20 *μ*g/kg/min or similar doses of other adrenergics except for forced optimisation of preload and afterload) [6]. In the current analysis, adrenaline, noradrenaline, dopamine, and dobutamine were modeled. Of all catecholamine variables, only noradrenaline doses exceeding 0.2 *μ*g/kg/min were different between Levels A and C; however, this was not true for Levels B and C (Table 2). This could be due to a selection process between the two Levels of A and B based on the assumption that a coordinator attempts to push a donor heart with very good functional and morphologic condition through the allocation process until heart transplantation.

### 4.2. Limitations and Strengths

Limitations of the current analysis include the fact that it concerns a secondary data analysis, where collection of the data variables is not tailored exactly to the research question [23], and the fact that the assumed ischemia time could not be analysed. Naturally, only the ischemia times of organs that were actually transplanted can be documented. If the decision not to accept an organ depends on expected ischemic time, this is not documented. However, in the practice of organ selection, organisational considerations play a considerable role in view of this variable. Recipient factors often influence the decision to accept a graft which may ordinarily be declined (e.g., older graft for older donor, marginal graft for sick HU recipient, good immunological match for highly sensitized patient, etc.). These factors are not documented at all or at least not in the database of the DSO and could not be analysed therefore.

The DSO data used in this analysis comprise a comprehensive data set that includes all potential donors in Germany during a time span of five years. The target population is therefore completely captured, and selection bias is limited to potential donors who were never registered in the DSO (external selection bias). A further strength of this study is the separation of the population into training and validation samples, so that results were checked within the same basic population. Between the training and validation samples, all models showed a high congruency in terms of the proportion of concordant pairs, and the different variable selection procedures (forward and backward) showed similar to identical results, thereby confirming the variables selected during model development based on the univariate results.

## 5. Conclusion

Thus, the current analysis clearly demonstrates that both guidelines of the ISHLT and German national recommendations (DTG) are more or less implemented in daily practice of heart donor evaluation in Germany. This study also showed that, within the donation process different donor, characteristics determine successful recovery of a heart for transplantation and that the impact of specific donor variables determine the fate of a graft at different decision levels. This should be taken in account by the implementation of binding recommendations or guidelines. Suboptimal evaluation of potential donor hearts result in a waste of resources with needless binding of capacities already in the run-up to transplantation and disappointing results which are difficult to explain to recipients and their relatives. Hence, standardisation of recommendations of the evaluation procedures for potential donor hearts and their mandatory implementation should be an issue of health policy.

## Key Points

- The current analysis is the first and exhaustive review of donor heart allocation process including all potential donors in Germany during a time span of five years.
- International and national guidelines are more or less implemented in daily practice of heart donor evaluation in Germany, but the impact of specific donor variables determines the fate of a graft at different decision levels of the allocation process.
- For health policy, standardisation of the evaluation procedures for potential donor hearts and their mandatory implementation should be promoted.

## Supplementary Material

Additional Supporting Information may be found in the online version of this article.
Table S1: List of variables included in the logistic regression model.
Table S2: Multivariate logistic regression results from the training sample and model-checking on the basis of the validation sample (Level A versus Level C):
High congruence in the proportion of concordant pairs.
The majority of variables in the models showed similar odds ratios and *p*-values. Changes from OR > 1 to OR < 1 or vice versa usually occurred in variables with wide confidence intervals and insignificant *p*-values, indicating that this might be due to random variation. This occurred in parameters as well as in categories representing missing values or results reported inadequately due to other more important pathologies (marked as “*??*”). Other categories of the same parameter showed congruent results of training sample and validation sample.
Table S3: Multivariate logistic regression results from the training sample and model-checking on the basis of the validation sample (Level B versus Level C):
High congruence in the proportion of concordant pairs.
The majority of variables in the models showed similar odds ratios and *p*-values. Changes from OR > 1 to OR < 1 or vice versa were usually occurred in variables with wide confidence intervals and insignificant *p*-values, indicating that this might be due to random variation. This occurred in parameters as well as in categories representing missing values or results reported inadequately due to other more important pathologies (marked as “*??*”). Other categories of the same parameter showed congruent results of training sample and validation sample.
Table S4: Explanations and abbreviations in Tables S2 and S3.

## Figures and Tables

**Table 1:** Characteristics of heart transplants in Germany 2006–2010 (German donors only).

Heart transplants, 2006–2010 (*n* = 1863)	Frequency	Percent
Recipient only received the heart	1728	92.8
Recipient received heart and other organs	135	7.3
Rescue allocation	301	16.2
Recipient at the highest urgency level (HU)	1460	78.4
Retransplantation	43	2.3
Matching gender between donor and recipient^a^	1272	68.3
Male donor/recipient female^a^	105	5.6
Female donor/male recipient^a^	485	26.0
Age of recipients (mean/median [25%/75% percentiles]) in years	46.5/51.0 [39/58]	
Age of donors (mean/median [25%/75% percentiles]) in years	39.0/43.0 [27/50]	

^a^Gender missing: *n* = 1.

**Table 2:** Multivariate logistic regression models comparing Level A versus C and Level B versus C.

Variable	Level A versus C (*n* = 2174: A = 1165; C = 1009)				Level B versus C (*n* = 1456: B = 447; C = 1009)			
	OR	95% CI		*P* value	OR	95% CI		*P* value
Age per year	0.87	0.85	0.89	<0.0001	0.95	0.94	0.96	<0.0001
Height per cm	1.04	1.02	1.06	<0.0001	1.02	1.02	1.03	<0.0001
ICU stay (per day)	1.40	1.21	1.61	<0.0001				
Cause of death (brain damage) secondary versus traumatic	0.21	0.09	0.50	0.0004				
Electrocardiogram: severe changes versus normal	0.05	0.01	0.26	0.0004	0.25	0.08	0.76	0.0150
Echocardiography left ventricular hypertrophy: severe (IVSd > 16 mm) versus none	0.20	0.09	0.49	0.0004	0.43	0.21	0.85	0.0154
Echocardiography left ventricular function: severely reduced (LVEF < 40%) versus good (LVEF ≥ 50%)	0.01	0.002	0.04	<0.0001	0.08	0.02	0.36	0.0008
Echocardiography heart valves: stenosis or insufficiency >1° versus normal	0.04	0.01	0.11	<0.0001	0.32	0.14	0.73	0.0072
Echocardiography wall motion abnormality: severe hypokinesia versus none	0.03	0.01	0.10	<0.0001	0.24	0.11	0.52	0.0003
Echocardiography wall motion abnormality: segmental akinesia versus none	0.03	0.004	0.21	0.0004	0.39	0.09	1.71	0.2116
History of arterial hypertension	0.36	0.20	0.63	0.0003				
Pneumonia	0.56	0.34	0.92	0.0213				
Hepatitis B					0.33	0.19	0.57	<0.0001
Hepatitis C	0.11	0.02	0.53	0.0060	0.07	0.03	0.15	<0.0001
Norepinephrine (most recent) >0.2 versus 0 *µ*g/kg/min	0.40	0.20	0.79	0.0091				
Blood transfusions	0.52	0.27	0.99	0.0448				
CK (creatine kinase) per 100 IU/L					0.99	0.99	0.10	0.0256
Troponin T or I: >0.2 versus <0.1 ng/mL	0.19	0.07	0.54	0.0018	0.27	0.14	0.51	<0.0001
LDH (lactate dehydrogenase) per 10 IU/L	0.98	0.98	0.99	0.0001				
Coronary artery disease known before donor evaluation	0.003	<0.001	0.01	<0.0001	0.03	0.01	0.09	<0.0001
Coronary angiography (if indicated) not performed versus normal	0.06	0.03	0.15	<0.0001	0.40	0.27	0.60	<0.0001

Blank lines indicate that the parameter was not relevant in the particular model. OR describes probability of heart transplantation when a risk factor exists compared to reference. There was a high concordance between study group and validation group. Therefore only data from study group data are shown here. IVSd: interventricular septal thickness at diastole; LVEF: left ventricular ejection fraction.
