# Supplementary material for: Current Practice of Heart Donor Evaluation in Germany: Multivariable Risk Factor Analysis Confirms Practicability of Guidelines
Source: J Transplant. 2013 Sep 30;2013:701854. doi: 10.1155/2013/701854 (PMC3806127; doi:10.1155/2013/701854)
Supplement: Supplementary file 1 — Additional Supporting Information may be found in the online version of this article. Table S1: List of variables included in the logistic regression model. Table S2: Multivariate logistic regression results from the training sample and model-checking on the basis of the validation sample (Level A versus Level C): High congruence in the proportion of concordant pairs. The majority of variables in the models showed similar odds ratios and p-values. Changes from OR > 1 to OR < 1 or vice versa usually occurred in variables with wide confidence intervals and insignificant p-values, indicating that this might be due to random variation. This occurred in parameters as well as in categories representing missing values or results reported inadequately due to other more important pathologies (marked as “??”). Other categories of the same parameter showed congruent results of training sample and validation sample. Table S3: Multivariate logistic regression results from the training sample and model-checking on the basis of the validation sample (Level B versus Level C): High congruence in the proportion of concordant pairs. The majority of variables in the models showed similar odds ratios and p-values. Changes from OR > 1 to OR < 1 or vice versa were usually occurred in variables with wide confidence intervals and insignificant p-values, indicating that this might be due to random variation. This occurred in parameters as well as in categories representing missing values or results reported inadequately due to other more important pathologies (marked as “??”). Other categories of the same parameter showed congruent results of training sample and validation sample. Table S4: Explanations and abbreviations in Tables S2 and S3. [file 701854.f1.docx]

|  | **Variable** | **Value (unit)** |
| --- | --- | --- |
|  | Age | Number (years) |
|  | Sex | m/f |
|  | Height | Number (cm) |
|  | BMI | Number (kg/m^2^) |
|  | ICU days | Number (Days since registration with DSO) |
|  | ICU days quadratic | Number ( Days^2^ since registration with DSO ) |
|  | Brain damage | a=atraumatic / t=traumatic / s=secondary |
|  | Cancer | Yes/Blank =not determined |
|  | Acute Meningitis | Yes/No |
|  | Pneumonia | Yes/No |
|  | Hepatitis B | Yes/No |
|  | Hepatitis C | Yes/No |
|  | Cytomegaly (anti-CMV IgG) | n=negative, p=positive, blank=not determined |
|  | Toxoplasmosis | n=negative, p=positive, blank=not determined |
|  | Acute Sepsis | Yes/No |
|  | Arterial hypertension | Yes/No |
|  | Diabetes | Yes/No |
|  | Coronary heart disease | Yes/No |
|  | Drug abuse | Yes/No |
|  | Smoking | Yes/No |
|  | Alkohol abuse | Yes/No |
|  | Chest trauma | 1=Yes/0=No |
|  | CK (creatine kinase) | Number (IU/l) |
|  | Troponin | Missing / normal (0≤value≤0.1 μg/l) / slightly elevated (0,1 <value≤ 2.0μg/l) / strongly elevated (>2.0μg/l) |
|  | GOT (glutamatic oxalacetic transaminase) | Number (IU/l) |
|  | LDH (lactate dehydrogenase) | Number (IU/l) |
|  | Hemoglobin | Number (μg /l) |
|  | Quick test (thromboplastin) | Number (%) |
|  | Noradrenaline at time of registration with ET | not administered / low dose (≤ 0,1μg/kg/min) / medium dose (0,1< Wert ≤0,2 μg/kg/min) / high dose (> 2 μg/kg/min) |
|  | Adrenaline at time of registration with ET | 1=Yes/0=No |
|  | Dobutamine at time of registration with ET | 1=Yes/0=No |
|  | Dopamine at time of registration with ET | 1=Yes/0=No |
|  | Plasma volume expander, admission until time of registration with ET | 1=Yes/0=No |
|  | Blood transfusion, admission until time of registration with ET | 1=Yes/0=No |
|  | Cardiopulmonary resuscitation , admission until time of registration with ET | 1=Yes/0=No |
|  | Grauhan ECG criteria | nr=normal / ER=elevated risk (bundle branch block, chronic VHF or Sokolow Lyon Index > 3.5cm, unifocal VES) / DER=distinctly elevated risk (additional ER due to infarction indications in the QRS complex, multifocal VES, pacemaker) |
|  | Left ventricular function in echo | ??=not discernable (cannot be judged) / norm=systolic normal / min red=minimally reduced (measured EF 40-49% or verbally described) / red=reduced (measured EF < 40% or verbally described) |
|  | Left ventricular hypertrophy in echo | missing=not determined / ??=not discernable (cannot be judged)/ pronounced=pronounced LVH / dil=dialated left ventricle without LVH / mild=moderate LVH / nr=normal |
|  | Valvular morphology in echo | ??=not discernable (cannot be judged) / nr=normal / min=1° insufficiency / relev=relevant valvular insufficiency (stenosis or insufficiency > 1°) |
|  | Wall motion abnormalities in echo | ??=not discernable (cannot be judged) / nr=normal / akin seg=regional akinesia / min hypokin=minimal global hypokinesia / rel hypokin=relevant global hypokinesia |
|  | CHD in coronary angiography (assuming 50% stenosis) | missing=not conducted / 0=no coronary vessel abnormalities / 0.5= wall irregularities or stenosis <50% / 1=single-vessel CHD (stenosis ≥50%) in the RCA, RCX, RIVA, or branches / 2=two-vessel or three-vessel CHD (stenosis ≥50%) affecting two or three of the RCA, RCX, RIVA, or branches |

**Table S1:** List of variables included in the logistic regression model

| **Decision level**  A (Heart allocation not initiated due to medical exclusion criteria) versus C (heart transplanted)  **Variable:** | Forward selection - Training sample  99.0% concordant pairs;  n=2,174 (A:1165, C:1009) | | | | Validation sample model  99.3% concordant pairs;  n=1,235 (A:694; C:541) | | | |
| --- | --- | --- | --- | --- | --- | --- | --- | --- |
|  | OR | 98% Confidence Interval | | p-value  (WCT) | OR | 98% Confidence Interval | | p-value  (WCT) |
| **Age (per year)** | 0.871 | 0.850 | 0.892 | <.0001 | 0.883 | 0.856 | 0.910 | <.0001 |
| **Height (per cm)** | 1.042 | 1.022 | 1.063 | <.0001 | 1.031 | 1.005 | 1.059 | 0.0213 |
| **ICU days (per day)** | 1.396 | 1.208 | 1.613 | <.0001 | 1.176 | 1.050 | 1.317 | 0.0051 |
| **ICU days (quadratic per day)** | 0.989 | 0.983 | 0.996 | 0.0010 | 0.995 | 0.993 | 0.998 | 0.0009 |
| **Sex f vs. m** | 1.922 | 1.113 | 3.319 | 0.0192 | 1.816 | 0.811 | 4.066 | 0.1466 |
| **Brain damage (atraumatic vs. traumatic)** | 0.527 | 0.264 | 1.050 | 0.0686 | 0.756 | 0.305 | 1.874 | 0.5455 |
| **Brain damage (s vs. t)** | 0.208 | 0.087 | 0.499 | 0.0004 | 0.151 | 0.047 | 0.486 | 0.0015 |
| **Grauhan ECG criteria (DER vs. nr)** | 0.048 | 0.009 | 0.260 | 0.0004 | 0.085 | 0.015 | 0.490 | 0.0058 |
| **Grauhan ECG criteria (ER vs. nr)** | 0.212 | 0.086 | 0.522 | 0.0007 | 0.393 | 0.088 | 1.753 | 0.2211 |
| **Grauhan ECG criteria (missing vs. nr)** | 0.090 | 0.049 | 0.166 | <.0001 | 0.131 | 0.058 | 0.294 | <.0001 |
| **Left ventricular hypertension in echo (missing vs. nr)** | 0.016 | 0.008 | 0.032 | <.0001 | 0.016 | 0.006 | 0.044 | <.0001 |
| **Left ventricular hypertrophy in echo (?? vs. nr)** | 3.006 | <.0001 | >999.99 | 0.9826 | 6.076 | 0.036 | >999.99 | 0.4911 |
| **Left ventricular hypertrophy in echo (pronounced vs. nr)** | 0.204 | 0.085 | 0.491 | 0.0004 | 0.075 | 0.023 | 0.248 | <.0001 |
| **Left ventricular hypertrophy in echo (dil vs. nr)** | 0.223 | 0.040 | 1.249 | 0.0878 | 0.012 | <0.001 | 0.706 | 0.0332 |
| **Left ventricular hypertrophy in echo (mild vs. nr)** | 0.478 | 0.234 | 0.974 | 0.0423 | 0.837 | 0.332 | 2.106 | 0.7047 |
| **Left ventricular function in echo (?? vs. norm)** | 0.027 | <.0001 | >999.99 | 0.9427 | 1.332 | 0.028 | 62.843 | 0.8840 |
| **Left ventricular function in echo (min red vs. norm)** | 0.852 | 0.267 | 2.717 | 0.7865 | 0.229 | 0.052 | 1.017 | 0.0526 |
| **Left ventricular function in echo (red vs. norm)** | 0.007 | 0.002 | 0.036 | <.0001 | 0.032 | 0.005 | 0.200 | 0.0002 |
| **Valvular morphology in echo (?? vs. nr)** | 0.832 | 0.037 | 18.910 | 0.9079 | 2.381 | 0.023 | 248.091 | 0.7144 |
| **Valvular morphology in echo (min vs. nr)** | 0.867 | 0.456 | 1.650 | 0.6648 | 1.372 | 0.592 | 3.180 | 0.4603 |
| **Valvular morphology in echo (relev. vs. nr)** | 0.037 | 0.013 | 0.105 | <.0001 | 0.109 | 0.024 | 0.490 | 0.0039 |
| **Wall motion abnormalities in echo (?? vs. nr)** | 6.697 | 0.126 | 357.01 | 0.3486 | 0.059 | <0.001 | 8.827 | 0.2684 |
| **Wall motion abnormalities in echo (akin seg vs. nr)** | 0.030 | 0.004 | 0.208 | 0.0004 | 0.020 | 0.002 | 0.271 | 0.0032 |
| **Wall motion abnormalities in echo (min hypokin vs. nr)** | 0.204 | 0.045 | 0.930 | 0.0400 | 0.471 | 0.083 | 2.664 | 0.3946 |
| **Wall motion abnormalities in echo (rel hypokin vs. nr)** | 0.031 | 0.010 | 0.098 | <.0001 | 0.053 | 0.008 | 0.363 | 0.0027 |
| **Arterial hypertension (yes vs. no)** | 0.355 | 0.202 | 0.625 | 0.0003 | 0.426 | 0.204 | 0.891 | 0.0235 |
| **Pneumonia (yes vs. no)** | 0.558 | 0.339 | 0.917 | 0.0213 | 0.570 | 0.291 | 1.115 | 0.1005 |
| **Hepatitis C (yes vs. no)** | 0.111 | 0.023 | 0.532 | 0.0060 | 0.052 | 0.004 | 0.741 | 0.0292 |
| **Norepinephrine at time of registration with ET (high dose vs. not administered)** | 0.395 | 0.196 | 0.794 | 0.0091 | 0.209 | 0.078 | 0.558 | 0.0018 |
| **Norepinephrine at time of registration with ET (medium dose vs. not administered)** | 0.727 | 0.348 | 1.521 | 0.3978 | 0.723 | 0.245 | 2.140 | 0.5586 |
| **Norepinephrine at time of registration with ET (low dose vs. not administered)** | 1.423 | 0.778 | 2.602 | 0.2519 | 0.614 | 0.268 | 1.408 | 0.2497 |
| **Blood transfusion** | 0.519 | 0.274 | 0.985 | 0.0448 | 0.441 | 0.198 | 0.982 | 0.0449 |
| **Troponin (slightly elevated vs. nr)** | 0.562 | 0.294 | 1.074 | 0.0812 | 0.473 | 0.201 | 1.112 | 0.0860 |
| **Troponin (missing vs. nr)** | 0.441 | 0.249 | 0.782 | 0.0051 | 0.482 | 0.217 | 1.069 | 0.0725 |
| **Troponin (strongly elevated vs. nr)** | 0.191 | 0.067 | 0.540 | 0.0018 | 0.256 | 0.051 | 1.277 | 0.0965 |
| **LDH (for each 10 IU/L)** | 0.983 | 0.975 | 0.992 | 0.0001 | 0.985 | 0.976 | 0.995 | 0.0024 |
| **Alcohol abuse** | 0.539 | 0.296 | 0.983 | 0.0438 | 1.062 | 0.459 | 2.455 | 0.8880 |
| **Coronary heart disease** | 0.003 | <0.001 | 0.013 | <.0001 | 0.004 | <0.001 | 0.046 | <.0001 |
| **CHD in coronary angiograph (missing vs. 0)** | 0.064 | 0.027 | 0.150 | <.0001 | 0.026 | 0.006 | 0.107 | <.0001 |
| **CHD in coronary angiograph (0.5 vs. 0)** | 1.611 | 0.341 | 7.600 | 0.5470 | 0.140 | 0.015 | 1.342 | 0.0882 |
| **CHD in coronary angiograph (1 vs. 0)** | 0.282 | 0.031 | 2.554 | 0.2605 | 13.757 | 0.199 | 949.916 | 0.2250 |
| **CHD in coronary angiograph (2 vs. 0)** | 0.053 | 0.002 | 1.419 | 0.0799 | 0.090 | 0.001 | 5.983 | 0.2608 |

***Table S2: Multivariate logistic regression results from the training sample and model-checking on the basis of the validation sample: Comparison of Level A in which heart donation was excluded by the OPO before registration of the donor for organ allocation at ET due to medical findings with level C in which a heart was transplanted. ORs are displayed for the relative odds that a heart will be transplanted if the examined parameter is present as compared to the reference value.*** There was a high congruence in the proportion of concordant pairs. The majority of variables in the models showed similar odds ratios and p-values. Green lines: Changes from OR > 1 to OR < 1 or vice versa were usually associated to variables with wide confidence intervals and insignificant p-values, indicating that this might be due to random variation. This occurred in parameters as well as in categories representing missing values or results reported inadequately due to other more important pathologies (marked as “??”). Other categories of the same parameter showed congruent results of training sample and validation sample.

**Table S3:**

| **Decision level**  B (Heart donation terminated after start of allocation procedure) versus C (heart transplanted)  **Variable:** | Forward selection - Training sample  80.0% concordant pairs;  n= 1,456 (B:447, C:1009) | | | | Validation sample model  78.6% concordant pairs;  n=851 (B:250; C:601) | | | |
| --- | --- | --- | --- | --- | --- | --- | --- | --- |
|  | OR | 98% Confidence Interval | | p-value  (WCT) | OR | 95% Confidence Interval | | p-value  (WCT) |
| **Age (per year)** | 0.949 | 0.938 | 0.961 | <.0001 | 0.955 | 0.941 | 0.970 | <.0001 |
| **Height (per cm)** | 1.024 | 1.015 | 1.033 | <.0001 | 1.017 | 1.005 | 1.029 | 0.0039 |
| **Grauhan ECG criteria (DER vs. nr)** | 0.247 | 0.080 | 0.762 | 0.0150 | 0.444 | 0.112 | 1.752 | 0.2462 |
| **Grauhan ECG criteria (ER vs. nr)** | 0.902 | 0.464 | 1.753 | 0.7600 | 0.842 | 0.368 | 1.928 | 0.6840 |
| **Grauhan ECG criteria (missing vs. nr)** | 0.631 | 0.394 | 1.011 | 0.0554 | 0.758 | 0.421 | 1.365 | 0.3558 |
| **Left ventricular hypertrophy in echo (missing vs. nr)** | 0.502 | 0.251 | 1.006 | 0.0519 | 0.672 | 0.246 | 1.839 | 0.4394 |
| **Left ventricular hypertrophy in echo (?? vs. nr)** | 0.427 | 0.010 | 19.011 | 0.6601 | 1.257 | 0.005 | 295.09 | 0.9346 |
| **Left ventricular hypertrophy in echo (pronounced vs. nr)** | 0.426 | 0.213 | 0.849 | 0.0154 | 0.635 | 0.247 | 1.636 | 0.3471 |
| **Left ventricular hypertrophy in echo (dil vs. nr)** | 0.520 | 0.174 | 1.550 | 0.2405 | 0.224 | 0.017 | 3.013 | 0.2595 |
| **Left ventricular hypertrophy in echo (mild vs. nr)** | 0.646 | 0.446 | 0.934 | 0.0203 | 0.838 | 0.525 | 1.336 | 0.4570 |
| **Left ventricular function in echo (?? vs. norm)** | 2.508 | 0.014 | 462.44 | 0.7297 | -- | -- | -- | -- |
| **Left ventricular function in echo (min red vs. norm)** | 0.484 | 0.243 | 0.964 | 0.0388 | 0.449 | 0.178 | 1.134 | 0.0902 |
| **Left ventricular function in echo (Red vs. norm)** | 0.083 | 0.019 | 0.357 | 0.0008 | 0.253 | 0.036 | 1.759 | 0.1649 |
| **Valvular morphology in echo (?? vs. nr)** | -- | -- | -- | -- | 0.867 | 0.075 | 10.040 | 0.9088 |
| **Valvular morphology in echo (min vs. nr)** | 1.050 | 0.745 | 1.480 | 0.7812 | 1.071 | 0.697 | 1.645 | 0.7541 |
| **Valvular morphology in echo (relev vs. nr)** | 0.318 | 0.138 | 0.733 | 0.0072 | 0.393 | 0.140 | 1.101 | 0.0756 |
| **Wall motion abnormalities in echo (?? vs. nr)** | 1.144 | 0.032 | 41.110 | 0.9414 | 0.366 | 0.003 | 39.598 | 0.6743 |
| **Wall motion abnormalities in echo (akin seg vs. nr)** | 0.389 | 0.088 | 1.711 | 0.2116 | 0.172 | 0.026 | 1.125 | 0.0662 |
| **Wall motion abnormalities in echo (min hypokin vs. nr)** | 0.255 | 0.114 | 0.569 | 0.0008 | 1.485 | 0.404 | 5.453 | 0.5514 |
| **Wall motion abnormalities in echo (rel hypokin vs. nr)** | 0.241 | 0.112 | 0.519 | 0.0003 | 0.155 | 0.048 | 0.495 | 0.0017 |
| **Hepatitis B (yes vs. no)** | 0.327 | 0.186 | 0.572 | <.0001 | 0.510 | 0.233 | 1.115 | 0.0916 |
| **Hepatitis C (yes vs. no)** | 0.069 | 0.033 | 0.148 | <.0001 | 0.063 | 0.021 | 0.190 | <.0001 |
| **CK (for each 100 IU/L)** | 0.994 | 0.989 | 0.999 | 0.0256 | 1.001 | 0.990 | 1.012 | 0.8935 |
| **Troponin (slightly elevated vs. nr)** | 0.632 | 0.449 | 0.887 | 0.0081 | 0.726 | 0.464 | 1.136 | 0.1605 |
| **Troponin (missing vs. nr)** | 1.127 | 0.794 | 1.599 | 0.5047 | 0.706 | 0.459 | 1.088 | 0.1145 |
| **Troponin (strongly elevated vs. nr)** | 0.271 | 0.143 | 0.514 | <.0001 | 0.235 | 0.102 | 0.538 | 0.0006 |
| **Coronary heart disease** | 0.034 | 0.013 | 0.089 | <.0001 | 0.041 | 0.011 | 0.152 | <.0001 |
| **CHD in coronary angiograph (. vs. 0)** | 0.404 | 0.270 | 0.603 | <.0001 | 0.463 | 0.269 | 0.795 | 0.0053 |
| **CHD in coronary angiograph (0.5 vs. 0)** | 0.712 | 0.377 | 1.344 | 0.2947 | 0.310 | 0.139 | 0.689 | 0.0041 |
| **CHD in coronary angiograph (1 vs. 0)** | 0.143 | 0.053 | 0.388 | 0.0001 | 0.140 | 0.041 | 0.480 | 0.0018 |
| **CHD in coronary angiograph (2 vs. 0)** | 0.160 | 0.025 | 1.004 | 0.0505 | 0.047 | 0.001 | 1.803 | 0.1002 |

***Table S3: Modeling results from the training sample and model-checking on the basis of the validation sample (comparison of the Level B in which heart donation was stopped after allocation process for a recipeitn was initiated by ET with the level C in which the heart was transplanted). ORs are displayed for the relative odds that a heart will be transplanted if the examined parameter is present as compared to the reference value.*** There was a high congruence in the proportion of concordant pairs. The majority of variables in the models showed similar odds ratios and p-values. Changes from OR > 1 to OR < 1 or vice versa were usually associated to variables with wide confidence intervals and insignificant p-values, indicating that this might be due to random variation. This occurred in parameters as well as in categories representing missing values or results reported inadequately due to other more important pathologies (marked as “??”). Other categories of the same parameter showed congruent results of training sample and validation sample.

**Table S4: Explanations and abbreviations in Tables S2 and S3**

| **Explanations and abbreviations in Tables S2 and S3:** | |
| --- | --- |
| X.XXX | Values that vary considerably between the training and validation samples are indicted in color. |
| WCT | Wald-Chi^2^-Test |
| Brain damage | a=atraumatic / t=traumatic / s=secondary |
| Grauhan ECG criteria | nr=normal / ER=elevated risk (bundle branch block, chronic VHF or Sokolow Lyon Index > 3.5cm, unifocal VES) / DER=distinctly elevated risk (additional ER due to infarction indications in the QRS complex, multifocal VES, pacemaker) |
| Left ventricular hypertrophy in echo | missing=not determined / ??=not discernable (cannot be judged) /  pronounced=pronounced LVH / dil=dialated left ventricle without LVH / mild=moderate LVH / nr=normal |
| Left ventricular function in echo | ??=not discernable (cannot be judged) / norm=systolic normal / min red=minimally reduced (measured EF 40-49% or verbally described) / red=reduced (measured EF < 40% or verbally described) |
| Valvular morphology in echo | ??=not discernable (cannot be judged) / nr=normal / min=1° insufficiency / relev=relevant valvular insufficiency (stenosis or insufficiency > 1°) |
| Wall motion abnormalities in echo | ??=not discernable (cannot be judged) / nr=normal / akin seg=regional akinesia / min hypokin=minimal global hypokinesia / rel hypokin=relevant global hypokinesia |
| Norepinephrine at time of registration with ET | not administered / low dose (≤ 0,1μg/kg/min) / medium dose (0,1< Wert ≤0,2 μg/kg/min) / high dose (> 2 μg/kg/min) |
| Troponin | Missing / normal (0≤value≤0.1 μg/l) / slightly elevated (0,1 <value≤ 2.0μg/l) / strongly elevated (>2.0μg/l) |
| CHD in coronary angiography (assuming 50% stenosis) | missing=not conducted / 0=no coronary vessel abnormalities / 0.5= wall irregularities or stenosis <50% / 1=single-vessel CHD (stenosis ≥50%) in the RCA, RCX, RIVA, or branches / 2=two-vessel or three-vessel CHD (stenosis ≥50%) affecting two or three of the RCA, RCX, RIVA, or branches |
